# Biosynthesis and characterization of silver nanoparticles using *Ochradenus arabicus* and their physiological effect on *Maerua oblongifolia* raised in vitro

**DOI:** 10.1038/s41598-020-74675-9

**Published:** 2020-10-16

**Authors:** Hassan O. Shaikhaldein, Fahad Al-Qurainy, Mohammad Nadeem, Salim Khan, Mohamed Tarroum, Abdalrhaman M. Salih

**Affiliations:** Botany and Microbiology Department, College of Science King Saud University, P. O. BOX 2455, Riyadh, 11451 Saudi Arabia

**Keywords:** Nanoparticles, Plant sciences

## Abstract

Silver nanoparticles (AgNPs) are presently the most commonly generated engineered nanomaterials and are found in a wide range of agro-commercial products. The present study was designed to synthesize AgNPs biologically using *Ochradenus arabicus* leaves and investigate their effect on the morphophysiological properties of *Maerua oblongifolia* raised in vitro. Physicochemical methods (ultraviolet–visible spectroscopy, Fourier transform infrared spectroscopy, and transmission electron microscopy were performed for characterization and for obtaining microphotographs of the AgNPs. Shoots of *M. oblongifolia* (2–3 cm) grown in Murashige and Skoog medium supplemented with different concentrations of AgNPs (0, 10, 20, 30, 40, or 50 mg L^−1^) were used. Following 6 weeks of in vitro shoot regeneration, the shoot number, shoot length, leaf number, fresh weight, dry weight, chlorophyll content, total protein, proline level, and antioxidant enzyme activities of the plants were quantified. We found that 20 mg L^−1^ AgNPs increased the shoot number, shoot length, fresh weight, dry weight, and chlorophyll content of the plants. The maximum total protein was recorded in plants that were administered the lowest dose of AgNPs (10 mg L^−1^), while high concentrations of AgNPs (40 and 50 mg L^−1^) increased the levels of proline and the enzymes superoxide dismutase and catalase. Our results indicate that green-synthesized AgNPs may be of agricultural and medicinal interest owing to their effects on plants in vitro.

## Introduction

Nanotechnology is a new approach in the field of agriculture. In recent years, rapid progress in the field of nanotechnology has enabled the synthesis of engineered nanoparticles (NPs) of different types, sizes, and morphologies^1^. Although many scientists have reported many methods for the manufacturing of metallic NPs, biological synthesis using microorganisms and plant extracts is simpler, less expensive, and more environmentally friendly as compared to physicochemical procedures^2^; further, the NPs generated using plants are more stable, less toxic, and biocompatible^3^.

The exposure of a plant to NPs may exert a considerable impact on the plant, such as increase in the metabolic rate and acceleration in germination, growth, and development. NPs also provide new mechanisms of plant protection, trigger antioxidant enzymes, and enhance plant regeneration^4,5^. Thus, NPs can be applied in agricultural settings to achieve superior plant growth and yield^6^. However, a comprehensive understanding of the role of biogenic NPs in plant physiology at the molecular level is still lacking^7^.

Many types of NPs have been increasingly used for plants, including silver, gold, zinc, copper, titanium, silicon, and magnesium NPs^8^. At present, silver NPs (AgNPs) are the most commonly generated engineered nanomaterials and are present in a wide range of agro-commercial products^9^. At certain concentrations in plant cell cultures, AgNPs are reported to play an essential role in improving growth, photosynthetic efficiency, chlorophyll content, and notable secondary metabolites production^10–12^.

*Maerua oblongifolia* is a rare plant found in Saudi Arabia that belongs to the family Capparaceae. It is used as an antimicrobial agent and is used for treating several health conditions, such as fever, stomach ache, skin infections, urinary calculi, diabetes mellitus, and abdominal colic^13^. Owing to overexploitation for fodder, food, timber, and medicinal purposes as well as its slow regeneration rate, wild populations of this plant are decreasing. Therefore, there is a serious need to enhance the regeneration of *M. oblongifolia* with micropropagation^14,15^. This can be achieved effectively with the application of NPs. Few studies have investigated the effect of AgNPs on the morphophysiological characteristics of plants. Therefore, the objectives of the present study were to synthesize AgNPs and investigate their effect on the regeneration, biomass, and antioxidant enzyme activities of *M. oblongifolia* raised in vitro*.*

## Materials and methods

### Synthesis of AgNPs

AgNPs were synthesized biologically using *Ochradenus arabicus*. Silver nitrate (AgNO_3_) and sodium dodecyl sulfate (SDS) were purchased from Sigma-Aldrich Chemical Corp. All the solutions were prepared in deionized Milli-Q water. *Ochradenus arabicus* was provided by the tissue culture lab of the King Saud University, Riyadh, Saudi Arabia. The *O. arabicus* plant extract that was used for the reduction of Ag^+^ to Ag^0^ was prepared by putting 5 g of thoroughly cleaned, finely chopped leaves in a round-bottom flask with 100 mL of deionized water and boiling the mixture for 10 min. Thereafter, the extract was filtered and kept in a refrigerator at 4 °C for use in further experiments. A 100-mL solution of 1 mM AgNO_3_ was prepared at room temperature. SDS was prepared by adding 1 mM of SDS to 100 mL of deionized water and used as a stabilizing agent. Finally, the AgNO_3_ mixture, SDS stabilizing agent, and leaf extract were mixed in a ratio of 2:1:2. The mixture was heated at 60 °C until its color changed from faint yellow to dark brown, indicating the AgNPs formation.

### Characterization of AgNPs

The biosynthesized AgNPs were characterized using different methods of analysis. First, ultraviolet–visible (UV–Vis) spectroscopy was performed to check the reduction technique used for AgNPs synthesis. Fourier transform infrared spectroscopy (FTIR) was used to monitor the presence of potential biomolecules and functional groups. X-ray diffraction was used to investigate the formation, crystalline behavior, and quality of the bioreduced AgNP powder. The shape and size of the synthesized AgNPs were assessed using transmission electron microscopy (TEM).

### Plant materials

Specimens of wild *M. oblongifolia* were collected from the southern parts of Saudi Arabia and multiplied in vitro via micropropagation in Murashige and Skoog (MS) media, as per the method described by Al-Qurainy et al.^15^. in the tissue culture laboratory of the King Saud University.

The experiment was performed in the tissue culture laboratory of the King Saud University. Different concentrations of AgNPs (0 mg L^−1^, 10 mg L^−1^, 20 mg L^−1^, 30 mg L^−1^, 40 mg L^−1^, and 50 mg L^−1^) were added to the MS media and *M. oblongifolia* nodal segments of 2–3 cm in length were transplanted into Magenta boxes (GA-7). Each box contained 50 mL of MS media as well as five explants. The tests for each treatment were conducted in triplicate.

Samples were collected after 45 days of culture to analyze the plant performance in terms of the following growth parameters: shoots number per explant, shoot length per explant, leaves number per explant, fresh weight, and dry weight.

### Estimation of chlorophyll content

The content of chlorophyll a (chl a) and chlorophyll b (chl b) in the leaves were estimated as per the methodology reported by Arnon^16^. For each treatment, 0.1 g of fresh leaves were weighed and then macerated in 80% acetone. The samples were stored at − 4 °C for 24 h before the mixture was transferred to a 2-mL Eppendorf tube. Finally, absorbance was read using a UV-1800 spectrophotometer (Shimadzu, Japan) at 663 nm (chl a) and 645 nm (chl b).

### Estimation of the total soluble protein content

The total soluble protein content was estimated using the method developed by Bradford^17^. Fresh leaves (0.3 g) were homogenized in 1-mL phosphate buffer. Equal volume of supernatant and TCA were mixed and centrifuged; the pellet was dissolved in 1 mL of 0.1 N NaOH. The absorbance was measured photometrically at 595 nm with bovine serum albumin as the standard. The protein content was expressed as mg g^−1^ of the fresh weight.

### Estimation of the proline content

The proline content was determined as per the method proposed by Bates et al.^18^. We homogenized 400 mg of fresh leaves in 10 mL of 3% aqueous sulfosalicylic acid. Thereafter, the mixture was centrifuged; 2 mL of supernatant was placed in a test tube, and 2 mL of ninhydrin and 2 mL of glacial acetic acid were added. Then, we heated the mixture at 100 °C for 1 h. After boiling, the reaction was stopped by placing the tubes in an ice bath for 5 min. Thereafter, 6 mL of toluene was added to each tube and mixed vigorously for 15 s. The absorbance of the upper phase was read at 520 nm using a UV-1800 spectrophotometer (Shimadzu, Japan). The proline content was expressed as μg g^−1^ fresh weight.

### Enzyme extraction and estimation of the enzyme activity

Enzyme extraction and measurement were performed as per the methods described by Jogeswar et al.^19^. *Maerua oblongifolia* leaves were initially ground in liquid nitrogen and dissolved in 100 mM sodium phosphate buffer (pH 7.4) that contained 0.1 mM ethylenediaminetetraacetic acid, 1% (w/v) polyvinylpyrrolidone, and 0.5% (v/v) Triton-X 100. The homogenous mixture was centrifuged at 10,000 rpm for 10 min at 48 °C to obtain the supernatant.

Superoxide dismutase (SOD, EC 1.15.1.1) activity was assessed using the method of Marklund and Marklund^20^. The reaction mixture contained 1 mL of 0.25 mM pyrogallol, 1.9 mL of 0.1 M sodium phosphate buffer (pH 7.4), and 100 μL of enzyme extract. The absorbance was measured at 420 nm. The SOD activity (U g^−1^ protein) was defined as the amount of enzyme needed for 50% inhibition of pyrogallol oxidation.

The catalase (CAT, EC 1.11.1.6) activity was recorded by measuring the absorbance at 240 nm, as per the method described by Claiborne^21^. The reaction mixture comprised 1 mL of 0.059 M H_2_O_2_ in 0.1 M sodium phosphate buffer (pH 7.4), 1.9 mL of distilled water, and 100 μL of enzyme extract. The CAT activity was expressed as unit g^−1^ of protein.

### Statistical analyses

A completely randomized experimental design was used. Statistical analyses were performed using one-way analysis of variance and comparison was done using Duncan's new multiple range test (P ≤ 0.05) in SPSS v. 20 for Windows.

## Results

### Green synthesis and characterization of AgNPs

**Figure 1:**
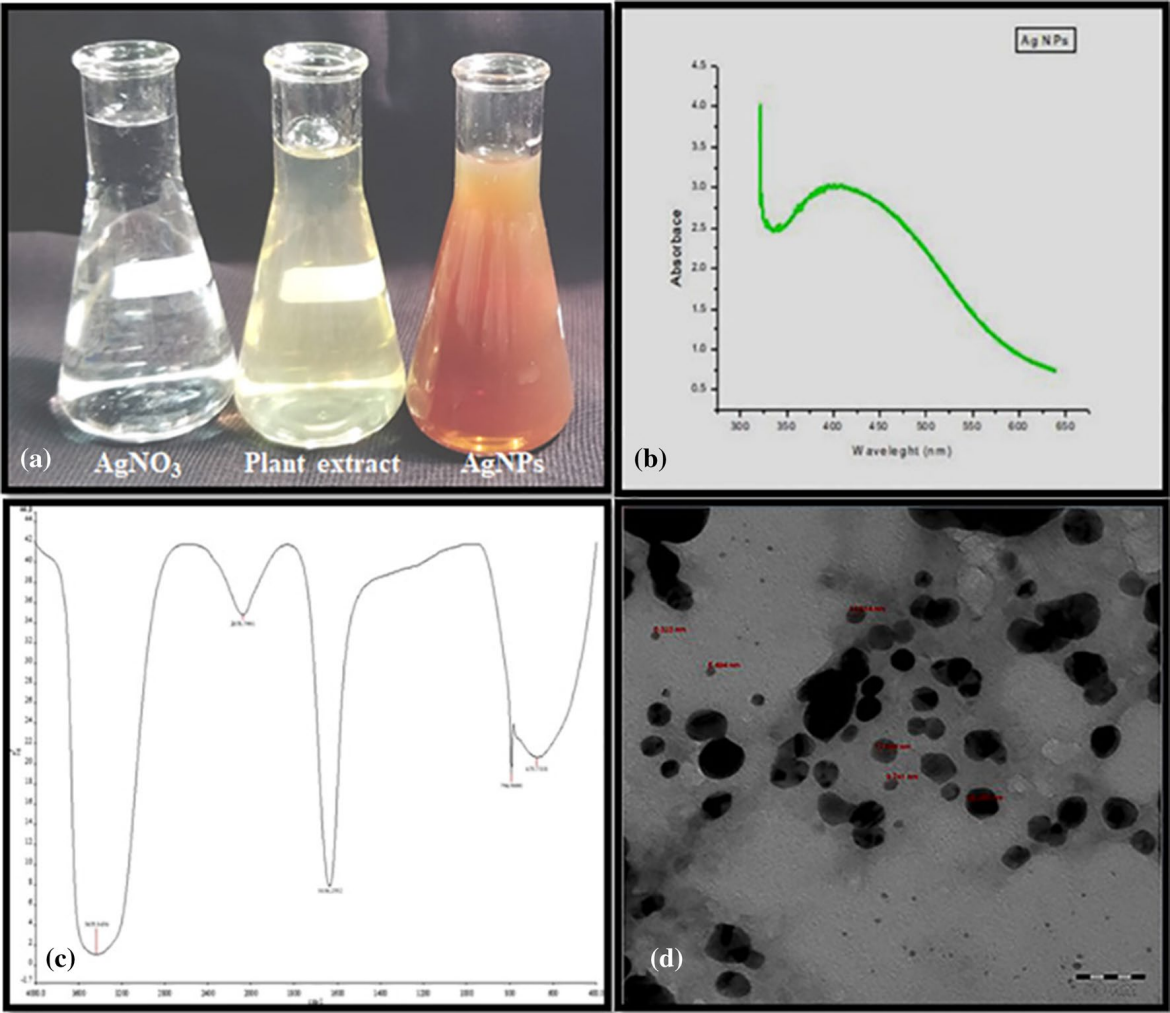
Silver nanoparticles formation (**a**). ultraviolet–visible absorption spectrum of the silver nanoparticles (AgNPs) with a plasmon band at 400 nm (**b**). Fourier transform infrared spectroscopy profile showing five peaks at 675 cm^−1^, 794 cm^−1^, 1634 cm^−1^, 2078 cm^−1^, and 3435 cm^−1^ (**c**). Transmission electron microscopy image of the AgNPs. Bar 50 nm (**d**).

AgNO_3_ reduction with *O. arabicus* leaves extract caused a visible color change on heating at 60 °C for 5 min; no color change was spotted in the control Ag solution Fig. 1a. This color change indicates AgNPs synthesis and is strongly connected to the surface plasmon resonance (SPR) of AgNPs^22^.

### UV–Vis spectroscopy

UV–Vis spectroscopy is an ideal technique for the characterization of AgNPs based on SPR^23^. The Ag SPR band showed a characteristic peak at 400 nm, as demonstrated in Fig. 1b.

### FTIR spectroscopy

The FT-IR profile showed five peaks at 675 cm^−1^, 794 cm^−1^, 1634 cm^−1^, 2078 cm^−1^, and 3435 cm^−1^, as shown in Fig. 1c. The shift in the peaks was obviously related to the reduction of Ag^+^ into AgNPs.

### Electron microscopy

The morphological analysis of the synthesized AgNPs was performed using TEM. The TEM micrograph illustrated that the synthesized AgNPs were spherical in shape and 6–24 nm in size, as shown in Fig. 1d.

### Effects of AgNPs on in vitro shoot reproduction

**Figure 2:**
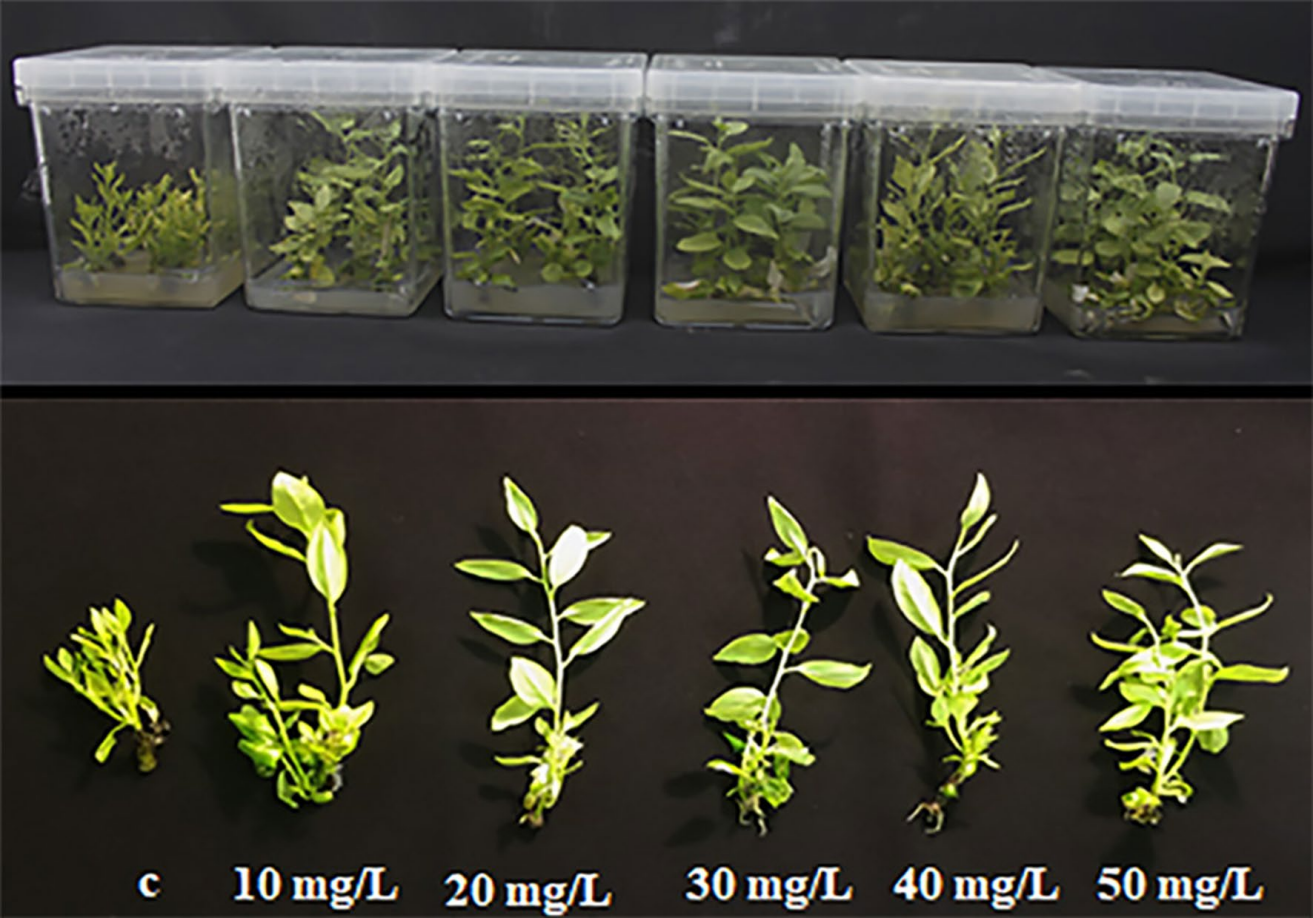
Influence of different concentrations of silver nanoparticles on the in vitro multiplication of (*Maerua oblongifolia*) after 45 days of culture in MS media. *C* control.

Different concentrations of AgNPs on in vitro *M. oblongifolia* showed significantly different effects on the morphological traits of the plants, such as shoot number, shoot length, fresh weight, dry weight, and leaf number (Fig. 2). All traits except leaf number showed significant differences with all treatments as compared to control (Table 1). Exposure to 20 mg L^−1^ AgNPs promoted the shoot length, fresh weight, and dry weight; exposure to 20 mg L^−1^, 30 mg L^−1^, and 40 mg L^−1^ AgNPs yielded the maximum number of shoots. However, the control group showed the least number of shoots and lowest shoot length as well as the lowest fresh and dry weights. There were slight differences in the leaf number among the treatments; higher concentrations of AgNPs (30 mg L^−1^, 40 mg L^−1^, and 50 mg L^−1^) increased the leaf number; the control group and plants treated with lower AgNPs concentrations (10 mg L^−1^ and 20 mg L^−1^) had the fewest leaves.

**Table 1:** Influence of the silver nanoparticles in the regeneration of *Maerua oblongifolia* after 45 days of treatment in MS Media.

Treatment (mg L^−1^)	Fresh weight (g)	Dry weight (g)	Shoot number (pot)	Shoot Length (cm)	Leaf number (pot)
0	2.33 ± 0.35^d^	0.37 ± 0.07^d^	8.67 ± 0.57^c^	3.43 ± 0.26^d^	178.00 ± 5.57^b^
10	5.40 ± 0.35^c^	1.17 ± 0.07^c^	13.33 ± 0.57^b^	8.90 ± 0.30^c^	174.00 ± 2.00^b^
20	7.46 ± 0.13^a^	1.57 ± 0.07^a^	16.67 ± 0.57^a^	10.43 ± 0.45^a^	167.33 ± 3.51^b^
30	6.57 ± 0.13^b^	1.43 ± 0.07^b^	16.33 ± 0.57^a^	9.73 ± 0.26^bc^	193.00 ± 3.00^a^
40	6.33 ± 0.05^b^	1.23 ± 0.07^c^	17.33 ± 0.57^a^	9.13 ± 0.26^c^	195.33 ± 2.52^a^
50	5.73 ± 0.27^c^	1.17 ± 0.07^c^	12.67 ± 0.57^b^	8.57 ± 0.32^c^	186.67 ± 5.03^a^

Data are the means of three replicates ± SD. The different letters “a”–“d” indicate significant differences between the treatments at P ≤ 0.05 according to the Duncan’s test.

### Effect of AgNPs on the chlorophyll content

**Figure 3:**
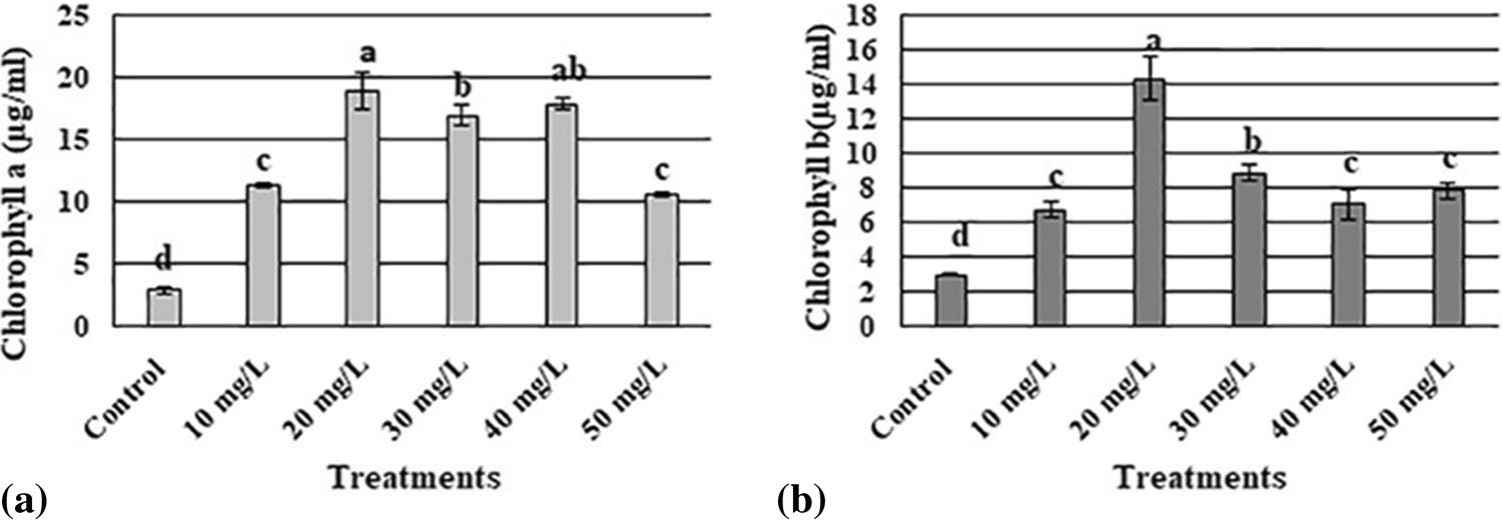
Influence of different concentrations of silver nanoparticles on the chlorophyll a (**a**) and chlorophyll b (**b**) content of *Maerua oblongifolia* after 45 days of treatment in MS Media. Data are the means of three replicates ± SD. The different letters “a”–“c” indicate significant differences between the treatments at P ≤ 0.05 according to the Duncan’s test.

The chl a and chl b, content of the plants differed significantly as per the AgNP concentrations to which the plants were exposed (Fig. 3). An AgNP concentration of 20 mg L^−1^ resulted in the highest levels of chl a and chl b, while the control group had the lowest chl a and chl b levels.

### Effect of AgNPs on total protein contents

**Figure 4:**
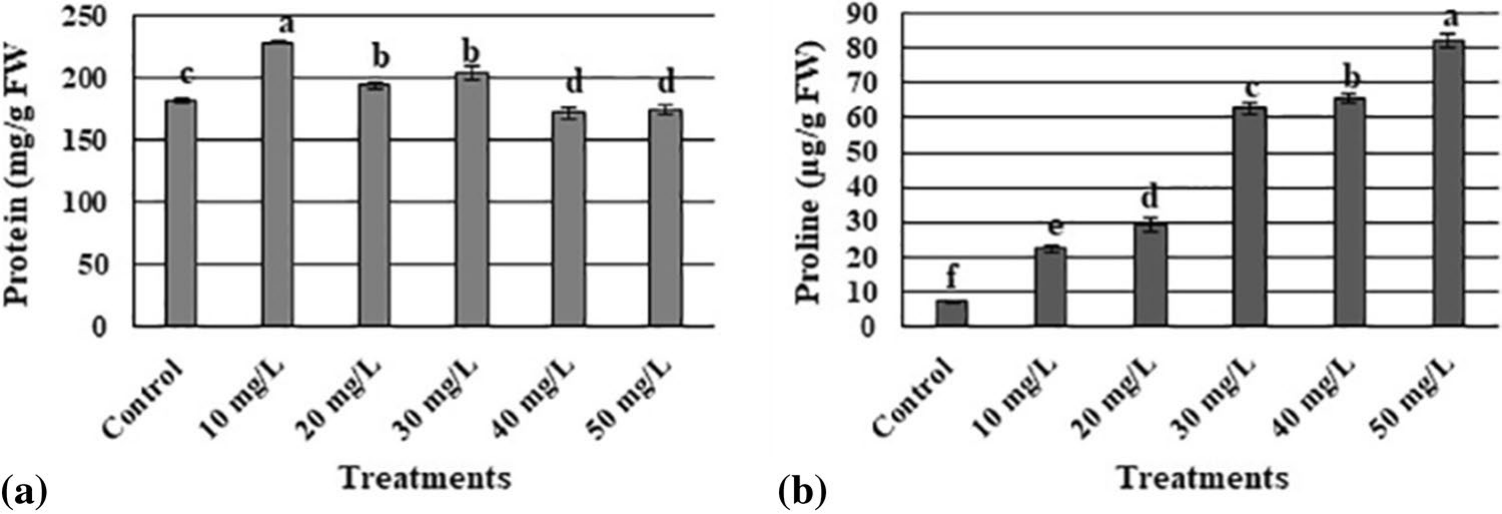
Influence of different concentrations of silver nanoparticles on protein (**a**) and proline (**b**) content in *Maerua oblongifolia* after 45 days of treatment in MS Media. Data are the means of three replicates ± SD. The different letters “a”’–“f” indicate significant differences between the treatments at P ≤ 0.05 according to the Duncan’s test.

There were significant differences in total protein contents among plants treated with different concentrations of AgNPs. The lowest concentration of AgNPs (10 mg L^−1^) resulted in the highest level of total protein, while high concentrations (40 and 50 mg L^−1^) resulted in the lowest total protein (Fig. 4 a).

### Effect of AgNPs on proline content

The results showed significant differences in proline content among all treatments. There was an exponential increase in the proline level as AgNP concentration increased. In general, high concentrations of AgNPs resulted in high proline levels. The highest proline level was in plants treated with 50 mg L^−1^ AgNPs, and the control group had the lowest proline contents (Fig. 4b).

### Effect of AgNPs on SOD and CAT activities

**Figure 5:**
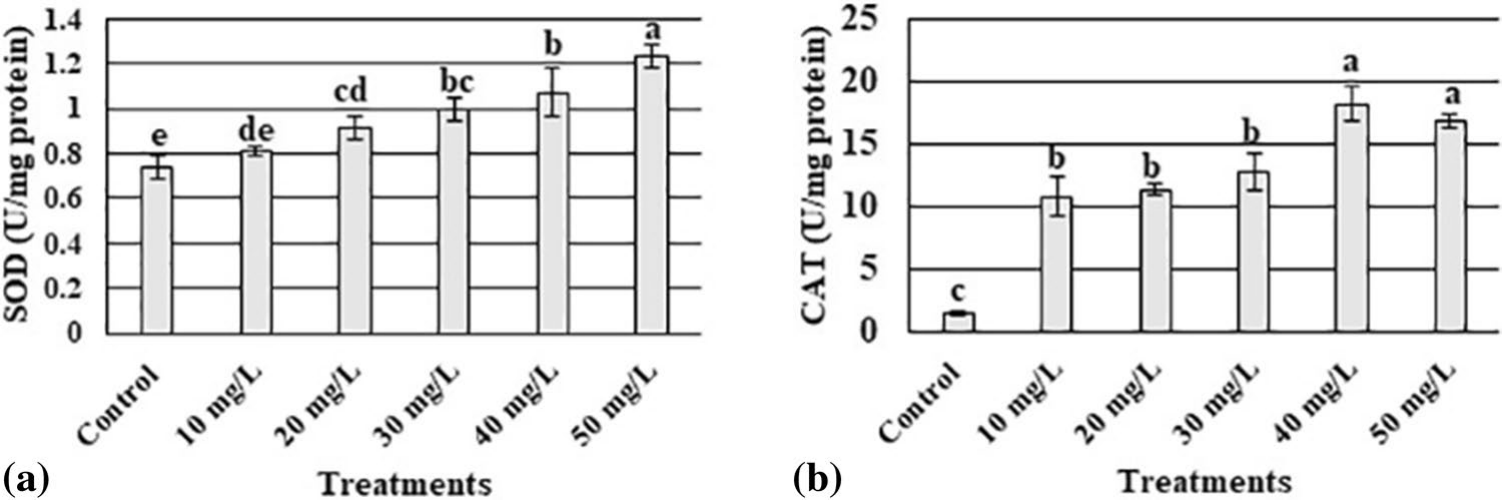
Influence of different concentrations of silver nanoparticles on superoxide dismutase (SOD) (**a**), and catalase (**b**) activities in *Maerua oblongifolia* after 45 days of treatment in MS Media. Data are the means of three replicates ± SD. The different letters “a”–“e” indicate significant differences between the treatments at P ≤ 0.05 according to the Duncan’s test.

We analyzed the SOD and CAT activities to determine the impact of AgNPs on the activities of enzymes that were related to oxidative stress. The activity of both the enzymes was stimulated in plants treated with high concentrations of AgNPs (40 mg L^−1^ and 50 mg L^−1^) (Fig. 5a,b).

## Discussion

In the present study, UV–Vis spectroscopy showed a peak at around 400 nm, typical in AgNPs, and indicated that the particles were dispersed without aggregation^22^. The FTIR results showed a band around 3440 cm^−1^, potentially resulting from OH stretching; meanwhile, the peak at 1634 cm^−1^, assigned to amide I, appears to be caused by carbonyl stretching in proteins^24^. The peak at around 800 cm^−1^ was attributed to C=CH_2_ and those at around 675 cm^−1^ were attributed to CH^25^. TEM was used to characterize the shape and size of AgNPs that were synthesized by the reduction of Ag^+^ using *O. arabicus* leaf extract*.* The TEM images obviously showed that the synthesized AgNPs were spherical, in line with previous results^26,27^. The dispersion properties of the spherical particles can vary as per their size, exact shape, and composition^28^.

Developmental parameters are very important for understanding the effectiveness of nanomaterials used for the plants. In the present study, applying AgNPs to the culture medium significantly enhanced the shoot number, shoot length, dry weight, and leaf number. Treatment with 20 mg L^−1^ AgNPs yielded the highest shoot number, shoot length, and dry weight; however, these parameters were decreased in plants that were treated with high concentrations of AgNPs (40 mg L^−1^ and 50 mg L^−1^). Similar findings have been reported by a study on wheat seedlings^29^. Other nanomaterials have demonstrated similar effects. For example, iron-based NPs enhanced the growth of maize at low concentrations (10 mg L^−1^); however, the growth was retarded at high concentrations (100 mg L^−1^)^30^. Different to our findings, early published results reported that AgNPs caused toxicity and decreased the developmental parameters in *Spirodela polyrrhiza*^31^. These paradoxical results are most likely to be caused by the differences in plant species. The response of the chlorophyll content (chl a and chl b) to AgNP treatment in the present study correlates with a previous report^29^ in which the total chl in wheat grown in vitro was significantly promoted after treatment with 25 mg L^−1^ AgNPs. Further, another study showed a remarkable increase in the chlorophyll content in *Stevia rebaudiana* after treatment with 25 mg L^−1^ AgNPs^26^. In general, the application of AgNPs reportedly increases the photosynthetic pigment amount in vanilla^32^ and sugarcane^33^. The reason for the increased chlorophyll content in our study could be the increases in nitrogen, magnesium, and iron concentrations in the plant tissues treated with AgNPs, given that these elements are associated with chlorophyll biosynthesis^26^. In contrast, decreased in chlorophyll content were also reported in *Arabidopsis thaliana* and rice^34,35^. These different findings are probably due to chemical synthesizing of AgNPs and differences in treatment time.

The use of low AgNPs concentrations elevated the total protein content of the plants. High AgNP concentrations caused a substantial reduction in the total protein content to levels lower than those in controls. One study reported an increase in the protein content of common bean and corn after treatment with low concentrations of AgNPs and a decreased content of total protein with higher concentrations of AgNPs^36^. These decreases in the protein content after treatment with high concentrations of AgNPs could be attributable to the toxic effect of AgNPs^37^.

Proline is an amino acid that acts as a non-enzymatic antioxidant that alleviates the adverse effects of reactive oxygen species. Its accumulation is important for adaptive (or hormetic) responses, such as the scavenging of reactive oxygen species and the function of metal chelators as signaling molecules in the plant defense mechanism^38,39^. In our study, we observed an increase in the total proline content in all the treatment groups. The highest concentration of AgNPs (50 mg L^−1^) resulted in the highest proline content. One study has reported increasing proline accumulation in the roots of *Oryza sativa* that was exposed to high concentrations of AgNPs (0.5 mg L^−1^)^35^. Moreover, higher proline accumulation was observed in *Solanum lycopersicum* after the application of both chemically and biologically synthesized AgNPs^40^. Meanwhile, the proline content reportedly increased in *Arabidopsis thaliana* with the use of high doses of copper oxide NPs (10 mg L^−1^ and 20 mg L^−1^)^41^. Increased accumulation of proline, as observed in the present study, could aim to protect the plants from increased oxidative stress under AgNP stress. Alternatively, as proline is a potential inhibitor of Programmed Cell Death (PCD)^42^, higher proline biosynthesis might lower PCD^43^.

Antioxidant enzymes, such as SOD and CAT, are major protective factors against oxidative damage and are activated in plants on exposure to AgNPs. Many studies have reported that antioxidant enzymes increased significantly after the use of AgNPs. In the current study, SOD and CAT levels were increased after treatment with higher concentrations of AgNPs (40 mg L^−1^ and 50 mg L^−1^). Similar to our findings, a previous trial reported that SOD and CAT activity were increased when castor seeds were exposed to higher concentrations (1000 mg L^−1^ and 2000 mg L^−1^) of AgNPs^44^. Other studies have also shown increases in SOD and CAT activities in different species treated with NPs^45,46^, However, decreased SOD and CAT activities were also reported in wheat and *Allium cepa* following AgNP application^43,47^. These contradictory results are most likely to be caused by the differences in treatment time; plant species; and doses, shapes, and sizes of the AgNPs.

## Conclusion

In sum, we successfully synthesized spherical AgNPs with size 6–24 nm with green synthesis using *O. arabicus* leaf extract. The biosynthesized AgNPs significantly improved the growth and development of *M. oblongifolia* propagated in vitro at both the morphological and physiological levels.

The application of 20 mg L^−1^ AgNPs to the culture media markedly enhanced shoot formation and increased the plant weight, chlorophyll content, and total protein content. Higher AgNPs concentration inhibited development, but increased the proline content and stimulated the production of antioxidant enzymes.

Our results indicate that the application of AgNPs to the in vitro culture media of plant tissues exerted positive effects; thus, green-synthesized AgNPs can be used for agricultural and medicinal purposes. However, further research is necessary for a clearer understanding of the molecular mechanism of AgNPs in cell developmental processes and the secondary metabolism.
